# Book Notices

**Published:** 1868-02-01

**Authors:** 


### *BOOK NOTICES.*

---

On the Signs and Diseases of Pregnancy. By Thomas Hawkes Tanner, M.D., F.L.S., etc. From the Second and Enlarged London Edition. With Four Colored Plates, and Illustrations on Wood. Philadelphia: Henry C. Lea. 1868. Pp. 490.

The imprint of this well-known publisher is almost of itself the guarantee of a good book, and when to it add the name of an author so eminent as is Dr. Tanner, it is about unnecessary to use language of commendation.

The JOURNAL thinks, however, that this is the best book which has thus far appeared on these important subjects. Clear, concise, practical, and to the point—we take unusual pleasure in commending it to our readers.

THE TREATMENT OF DISEASES OF THE THROAT AND LUNGS BY INHALATIONS, with a New Inhaling Apparatus. By EMIL SIEGLE, M.D. Translated from the Second German Edition by S. Nickles, M.D., Cincinnati. R. W. Carroll & Co., Publishers. 1868. Pp. Price $1.25. Sent to any part of the United States.

This is a very interesting and valuable little book, which should be read by all those who are employing "pulverized fluids" in the treatment of affections of the air passages. Its practical suggestions are given in a lucid and rational manner. We are especially pleased by perusal of the sections on membranous croup, whooping-cough, and phthisis pulmonalis. In the latter affection, the author recognizes the prime importance of attention to hygiene and nutrition, but considers inhalations valuable adjuvants in relieving subordinate symptoms.

PLASTICS. A New Classification and a Brief Exposition of Plastic Surgery. A Reprint from a Report in the Transactions of the Illinois State Medical Society. By DAVID PRINCE, M.D. Philadelphia: Lindsay & Blakiston.

CHRONIC DISEASES OF THE LARYNX, with Special Reference to Laryngoscopic Diagnosis and Local Therapeutics. By Dr. ADELBERT TOBOLD, Lecturer in the University of Berlin. Translated from the German, and edited by GEORGE M. BEARD, A.M., M.D., Lecturer on Nervous Diseases in the University of New York. With an Introduction on the History and Art of Laryngoscopy, etc., etc., by the Editor. Illustrated by 44 engravings on wood. New York: Wm. Wood & Co, 61 Walker Street; London: Robert Hardwicke. 1868.

Condie on Diseases of Children.—The sixth revised and enlarged edition was announced on page 23 of the Journal. It may well be said that the great aim of the author "to present a complete and faithful exposition of the pathology and therapeutics of the maladies incident to the earlier stages of existence" has been faithfully followed up. Whilst upon some points the therapeutics of the book do not quite suit us, the pathology is mainly up to the times. We shall take an early opportunity to set forth, especially, our objections to the management of Diphtheria recommended. We are fully of the opinion that the plan of this author can not be endorsed by the profession of the North-west.

